# Ten-year follow-up of a total knee prosthesis combining multi-radius, ultra-congruency, posterior-stabilization and mobile-bearing insert shows long-lasting clinically relevant improvements in pain, stiffness, function and stability

**DOI:** 10.1007/s00167-022-07216-8

**Published:** 2022-11-06

**Authors:** Killian Cosendey, Antoine Eudier, Nicole Fleury, Luis C. Pereira, Julien Favre, Brigitte M. Jolles

**Affiliations:** 1 Swiss BioMotion Lab, Department of Musculoskeletal Medicine, Lausanne University Hospital and University of Lausanne, CH-1011 Lausanne, Switzerland; 2 Service of Orthopaedics and Traumatology, Broye Intercantonal Hospital, Payerne, Switzerland; 3 Operational Unit, Department of Musculoskeletal Medicine, Lausanne University Hospital and University of Lausanne, Lausanne, Switzerland; 4 The Sense Innovation and Research Center, Sion, Lausanne, Switzerland; 5 Institute of Electrical and Micro Engineering, Ecole Polytechnique Fédérale de Lausanne, Lausanne, Switzerland

**Keywords:** Knee, Osteoarthritis, Arthroplasty, Revision rate, Loosening, Questionnaires, Radiography, Gait analysis, Minimum important change

## Abstract

**Purpose:**

There is a paucity of data available on total knee prostheses combining dual-radius, ultra-congruency, posterior-stabilization and mobile-bearing insert. This prospective cohort study aimed to assess the clinical evolution of the FIRST^®^ prosthesis *(Symbios Orthopédie*, Yverdon, CH), the earliest prosthesis with this particular design. It was hypothesized that the primary outcomes, evaluating pain, stiffness, function and stability, would improve following arthroplasty and remain improved during the follow-up period of 10 years.

**Methods:**

All patients programmed for a total knee arthroplasty using a FIRST^®^ prosthesis at our university hospital between 2006 and 2008 were invited to participate. Study knees were evaluated pre-operatively as well as one, five and ten years post-operatively. Patients filled out questionnaires at each evaluation point and had a radiographic assessment at the five-year and ten-year follow-ups. Primary outcomes were the total, pain, stiffness and function measures of the Western Ontario and McMaster Universities Osteoarthritis questionnaire (WOMAC) and the knee and function measures of the Knee Society Score (KSS). Friedman and Wilcoxon’s rank-sum tests were used to compare measures across time points.

**Results:**

Hundred and twenty four prostheses were included (baseline demographics: 69.9 ± 8.3 years old, 28.1 ± 4.3 kg/m^2^, 54% male) and 68 could be followed during ten years. Five prostheses underwent a revision. All other prostheses lost at follow-up were lost for reasons unrelated to the prosthesis. All primary measures reported statistically and clinically significant improvements between baseline and the three follow-up evaluations. Statistically significant improvements at the three follow-up evaluations were also observed for most secondary measures. There was no implant loosening. At ten-year follow-up, radiolucent lines were observed in 2.9% femoral implants and 1.5% tibial implants.

**Conclusion:**

The positive results observed in all domains of assessment and the small revision rate demonstrated an effective functioning of the FIRST^®^ prosthesis during the ten-year follow-up period. The results, globally similar to those previously published for other prosthesis designs, encourage the development of assistive methods to select the most appropriate designs on an individual basis.

**Level of evidence:**

IV, prospective cohort study.

**Supplementary Information:**

The online version contains supplementary material available at 10.1007/s00167-022-07216-8.

## Introduction

A total knee prosthesis with an original design combining dual-radius, ultra-congruency, posterior-stabilization and mobile-bearing insert was launched in 2006 (*FIRST*^*®*^*, Symbios Orthopédie*, Yverdon, CH). More specifically, this prosthesis uses a two radius concept, with one axis offering ultra-congruency for flexion up to 90° and another one conferring posterior stabilization past 90° of flexion. Moreover, the femoral cam was designed to contact the tibia post past 90° of flexion, when the insert contacts the femur on the second, smaller radii. This feature allows the femur to roll back, which should facilitate deep knee flexion and reduce wear [8]. In addition, the FRIST^®^ prosthesis offers free rotation of the insert in the transverse plane without translation to prevent the paradoxical anterior femoral sliding, therefore reducing a potential source of wear and pain [9].

With over a decade of use, it is possible and important to assess the long-term clinical outcomes of the FIRST^®^ prosthesis. Doing so seems especially relevant as more than 50,000 implantations were performed this past decade in Europe alone (manufacturer’s numbers). Evaluating the long-term evolution of the FIRST^®^ prosthesis could also reveal pertinent for the field in general. Indeed, other prostheses adopted a similar design [16, 17, 30]. But, to our knowledge, no long-term evaluation has been published for these prostheses either.

This prospective cohort study aimed to assess the long-term clinical evolution of the FIRST^®^ prosthesis. It was hypothesized that the primary outcomes, evaluating pain, stiffness, function and stability, would improve by clinically relevant magnitudes following arthroplasty and remain improved during the follow-up period of ten years.

## Patients and methods

### Patients

All patients programmed for a total knee arthroplasty following primary osteoarthritis using a FIRST^®^ prosthesis at our university hospital between 2006 and 2008 were invited to participate in this monocentric prospective cohort study. All patients willing to participate were included without further screening. Patients could be included twice; once for each knee. All patients provided written informed consent before taking part in this research. All procedures were done in accordance with the local ethics committee and with the 1964 Helsinki declaration and its later amendments.

**Fig. 1:**
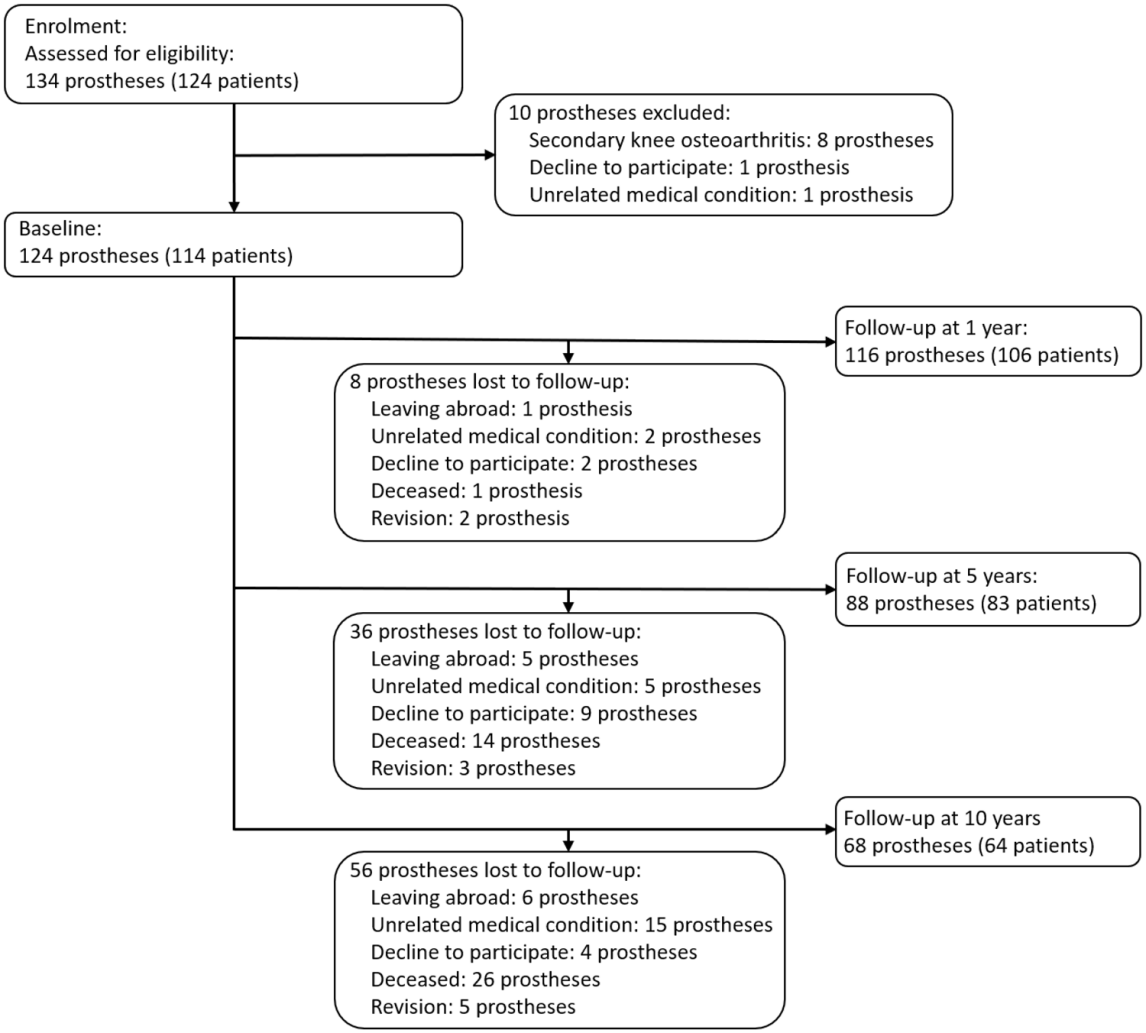
Flow diagram of the study prostheses

Out of the 134 FIRST^®^ prostheses consecutively implanted during the recruitment period, 124 were included in the present study (Table 1). Five prostheses were revised during the ten-year follow-up period, none for loosening (Fig. 1). One prosthesis was revised following a 180° insert rotation and dislocation six weeks after implantation, another one was revised following a severe knee torsion and post-traumatic pain one year after implantation, two prostheses were revised for debridement and mobile insert replacement following arthrofibrosis two years after implantation, and finally one prosthesis was revised for an extensor mechanism rupture with patellar comminuted fracture six years after implantation.

**Table 1:** Patient demographics for the 124 prostheses at baseline

Age (year)	69.6 ± 8.3
Men/Women (*n*)	67 / 57
BMI (kg/m^2^)	28.1 ± 4.3
Height (m)	1.7 ± 0.1
Weight (kg)	80.6 ± 15.9

Demographics are presented as mean ± SD or as number

*BMI* body mass index

### Surgical procedure

All arthroplasties in this study were performed by one of the three senior surgeons of the institution with more than 10 years of experience in total knee arthroplasty. The same procedure was used with all patients: antibiotic prophylaxis (intravenous cefuroxime 30 min pre-operatively and six hours post-operatively), tourniquet control and a medial para-patellar approach. A tibia-first technique was used in all cases. Bone cutting was performed with standard instrumentation. All prostheses were fully cemented after pulsatile lavage of the resected surfaces. The patella was resurfaced in all cases. Soft-tissue balancing aimed to restore the original alignment of the limb. Thrombo-prophylaxis, including low-molecular-weight heparin, was administrated the night before surgery for a duration of 6 weeks. All patients followed the same postoperative pain management protocol. Rehabilitation started one day after surgery with active full-weight bearing and passive physiotherapy. Crutches were recommended for 6 weeks.

### Methods of assessment

Study knees were evaluated pre-operatively (baseline) as well as one (1 yr), five (5 yr) and ten (10 yr) years post-operatively. Patients were invited to participate at the three follow-up evaluations, regardless of their participation in the previous follow-up assessments. The evaluations were done by two independent observers: one orthopedic surgeon and one physiotherapist used to clinical and radiological examination of the lower extremity. During each evaluation, patients filled up the Western Ontario and McMaster Universities Osteoarthritis questionnaire (*WOMAC*, range 0–100%, with 0% indicative of the best condition) [4], the Knee Society Score (*KSS*, range 0–100, with 100 indicative of the best condition) [20], the EuroQol five-dimensions questionnaire (*EQ-5D questionnaire*, range 0–1, with 1 indicative of the best condition) [13] and the EuroQol Visual Analog Scale (*EQ-5D VAS*, range 0–100, with 100 indicative of the best condition) for the index knee. The maximum passive flexion and extension angles as well as the neutral flexion–extension angle measured for the index knee with a goniometer while the patients were lying supine, were also documented. Measures for which a minimum important change (MIC) with respect to total knee arthroplasty was available in literature were considered primary outcomes. This was the case for *WOMAC total, pain, stiffness* and *function* measures, with MIC of 17%, 21%, 13% and 16%, respectively, as well as for *KSS knee* and *function* measures, with MIC of 9 and 10 units, respectively [10, 28].

**Fig. 2:**
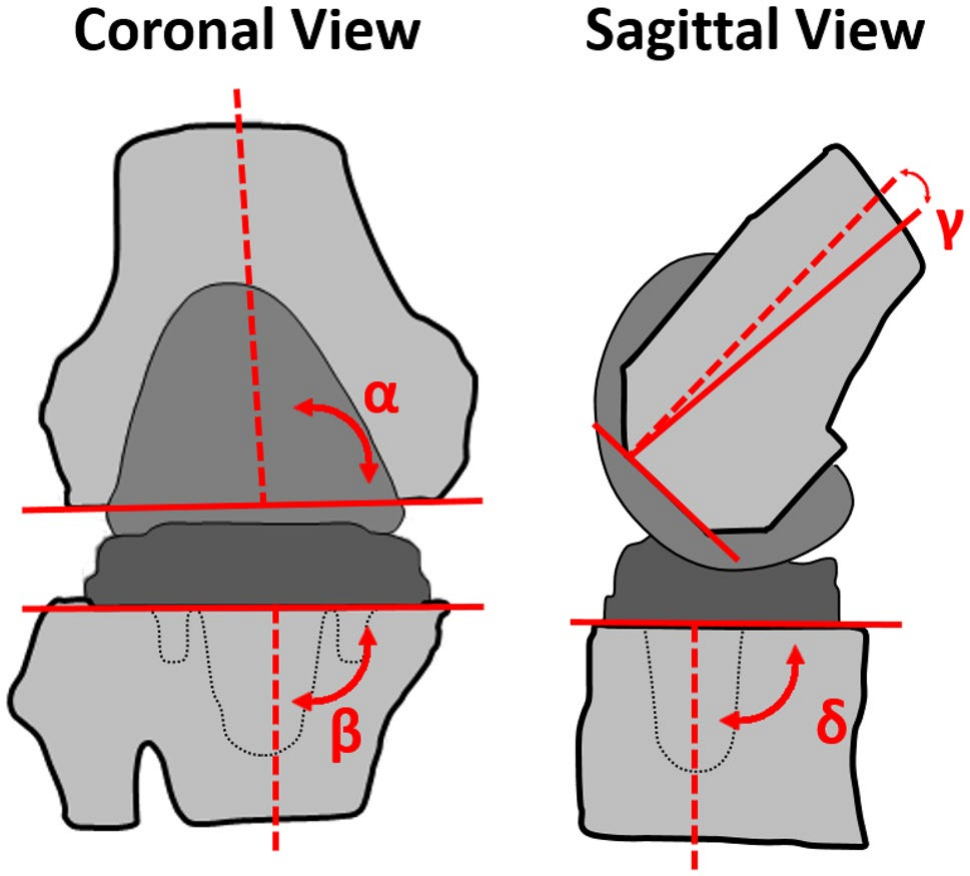
Radiographic measures of implants positioning. Coronal view. α: Femoral angle (°). β: Tibial angle (°). Sagittal view. γ: Femoral angle (°). δ: Tibial angle (°). Dashed lines represent the anatomical axes of the segments and solid lines represent the implant axes

The follow-up also included a radiographic evaluation at 5 yr and 10 yr. A coronal and a sagittal view, both at 30° of knee flexion, as well as a skyline patellar view were collected at both time points. The position of the tibial and femoral components (α, β, γ and δ angles) (Fig. 2) and the presence of radiolucent lines, defined as gaps of more than 2 mm between the bone and cement, were documented according to the Knee Society recommendations [3, 15]. Radiographic data were available for all but one prosthesis evaluated at 5 yr and for all prostheses assessed at 10 yr.

Furthermore, a gait analysis was performed at 1 yr and 5 yr. It consisted of two 30-m trials analyzed using an ambulatory device composed of inertial sensors fixed on the pelvis, thighs and shanks (Physilog^®^, BioAGM, Switzerland). The operating details and validation of this gait analysis method were published previously [11]. Walking speed; cycle, stance and double support durations; stride length; limp; as well as thigh, knee and shank ranges of motion (ROM) were measured for the index knee. Gait data were obtained for 83 prostheses at 1 yr and 71 prostheses at 5 yr, corresponding to 72% and 80% of the prostheses assessed at these time points, respectively.

### Statistical analysis

Since the measures followed non-normal distributions, they were reported as median and quartiles. Friedman and Wilcoxon’s rank-sum tests with Bonferroni correction were used to compare the measures across time points. The significance level was set a priori to 5%. A sample size calculation indicated that at least 32 prostheses were necessary to detect minimum important changes (MIC) in primary outcomes between evaluation time points with a power of 80% [7].

Additional Wilcoxon’s rank-sum tests indicated that three measures at baseline differed statistically significantly between the patients completing the 10 yr evaluation and the patients lost during follow-up. Specifically, the patients lost during follow-up were older (median difference of 7 years, p < 0.001), had a better *WOMAC pain* (median difference of 12.5%, p = 0.03) and a worse *KSS function* (median difference of 12.5 units, p = 0.005) at baseline. Data processing and statistical analyses were done with MATLAB^®^ software (MathWorks, MA).

## Results

**Fig. 3:**
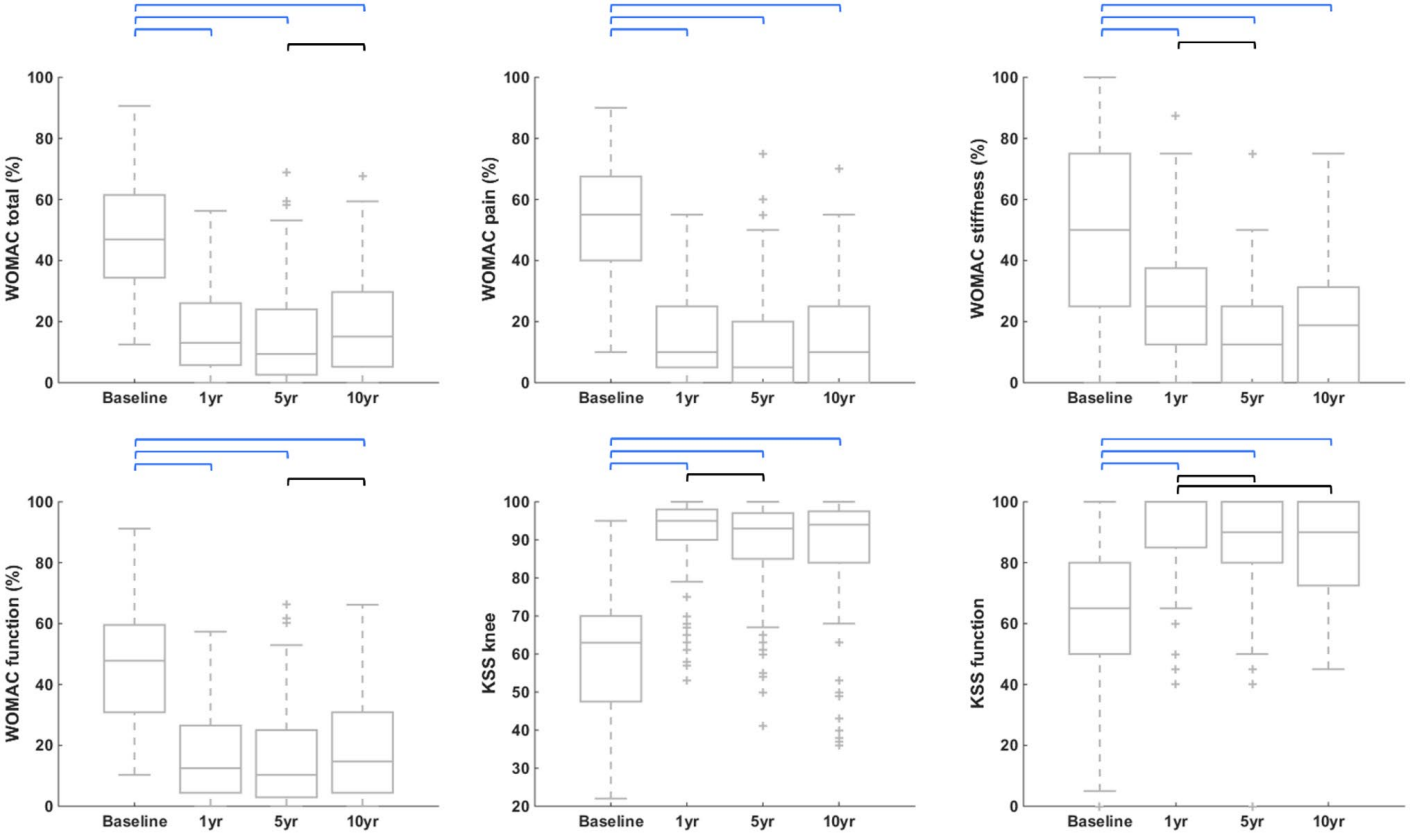
Boxplots of the primary measures at each time point. *WOMAC* Western Ontario and McMaster Universities Osteoarthritis Index, *KSS* Knee Society score. 1 yr, 5 yr and 10 yr: one-, five- and ten-year follow-ups, respectively. Black horizontal bars indicate statistically significant changes between time points, whereas blue horizontal bars indicate changes that were both statistically and clinically significant. Actual numbers are provided in Supplementary Materials 1

All primary measures reported statistically and clinically significant improvements between baseline and the three follow-up evaluations (1 yr, 5 yr and 10 yr) (Fig. 3 and Supplementary Material 1). Statistically significant changes were also observed between follow-ups, but they were below the minimum important change (MIC).

**Fig. 4:**
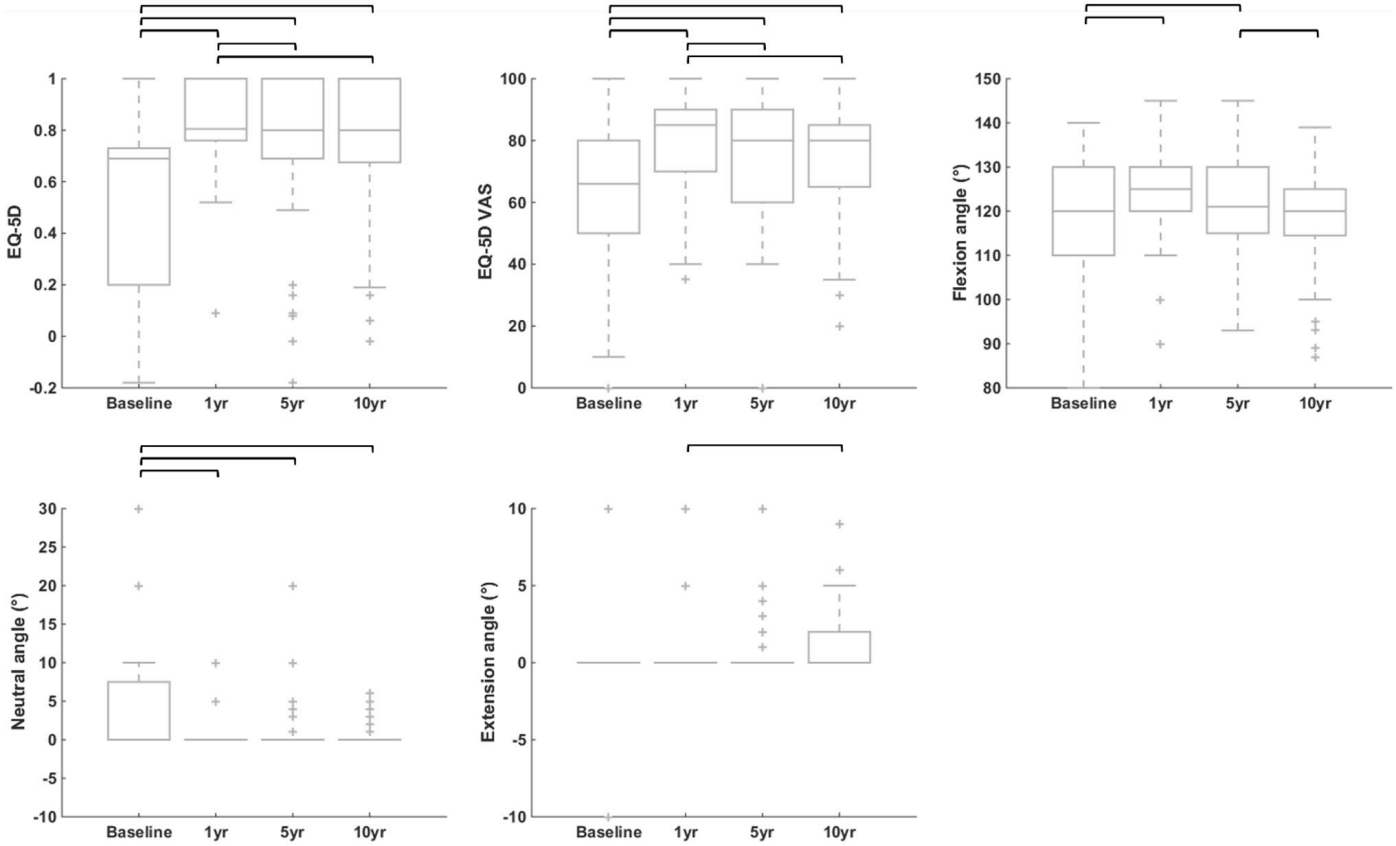
Boxplots showing the evolution of EuroQol quality of life (EQ-5D) and passive flexion–extension knee angles over time. 1 yr, 5 yr and 10 yr: one-, five- and ten-year follow-ups, respectively. Black horizontal bars indicate statistically significant changes between time points. Actual numbers are provided in Supplementary Materials 2

*EQ-5D questionnaire* and *VAS* were statistically significantly better at the three follow-up evaluations compared to baseline (Fig. 4 and Supplementary Material 2). Statistically significant improvements with respect to baseline were also observed for the maximum passive knee flexion angle at 1 yr and 5 yr and the neutral flexion angle at the three follow-up evaluations. Statistically significant changes among follow-up evaluations were also observed for some of these measures.

There was no implant loosening nor radiolucent line at 5 yr. At 10 yr, there was again no implant loosening, but radiolucent lines associated with mild pain were reported for two femoral implants and one tibial implants. This corresponded to 2.9% and 1.5% of the prostheses assessed at 10 yr, respectively. The three coronal angles (α, β and α + β) and the femoral sagittal angle (γ) changed statistically significantly between 5 and 10 yr, indicating that the knees became more valgus and the femoral implant more flexed (Table 2).

**Table 2:** Radiographic measures five (5 yr) and ten (10 yr) years after surgery, with their evolutions between time points

Measures	Time points		Evolution
	5 yr *n* = 87	10 yr *n* = 68	10–5 yr *n* = 66
Coronal view			
Femoral angle α (°)	92.4 [87.1; 94.3]	94.2 [92.6; 96.0]	**1.5 ** **[**− **0.4; 7.4]**
Tibial angle β (°)	88.5 [87.7; 89.4]	89.2 [87.8; 90.0]	**0.4 ** **[**− **1.0; 2.0]**
Total valgus angle α + β (°)	180.7 [175.9; 182.8]	182.8 [181.4; 185.2]	**2.6 ** **[0.3; 8.2]**
Sagittal view			
Femoral angle γ (°)	2.1 [3.3; 1.2]	1.1 [− 0.6; 2.3]	− **1.3 ** **[**− **2.5; 0.3]**
Tibial angle δ (°)	86.0 [84.6; 88.0]	86.9 [85.3; 88.3]	0.2 [− 1.2; 2.3]

Data are reported as median [25th percentile; 75th percentile]

*n* number of prostheses

Statistically significant changes between time points are bolded (*p* ≤ 0.05)

At 5 yr, patients walked statistically significantly slower than at 1 yr (Table 3). This occurred along with statistically significantly shorter stride length, decreased knee and shank ROM, as well as higher limping.

**Table 3:** Gait measures one (1 yr) and five (5 yr) years after surgery, with their evolutions between time points

Measures	Time points		Evolution
	1 yr *n* = 83	5 yr *n* = 71	5–1 yr *n* = 58
Speed (m/s)	1.2 [1.0; 1.3]	1.1 [1.0; 1.2]	− **0.1** **[**− **0.2; 0.0]**
GCD (s)	1.1 [1.1; 1.2]	1.1 [1.0; 1.2]	0.0 [0.0; 0.0]
Stride length (m)	1.3 [1.2; 1.4]	1.2 [1.1; 1.3]	− **0.1** **[**− **0.1; 0.0]**
Stance duration (%GCD)	60.2 [59.1; 61.6]	59.9 [58.2; 61.8]	− 0.9 [− 1.6; 0.9]
Double support duration (%GCD)	20.3 [18.2; 23.3]	19.9 [16.3; 23.6]	− 1.7 [− 3.2; 1.9]
Limp (%GCD)	1.6 [1.1; 2.3]	2.5 [1.6; 4.0]	**0.6** **[**− **0.2; 1.7]**
Thigh ROM (°)	41.2 [37.2; 45.9]	40.2 [38.0; 44.9]	− 0.3 [− 4.0; 2.5]
Knee ROM (°)	56.2 [51.9; 60.9]	49.5 [45.8; 54.7]	− **4.9** **[**− **10.2;** − **0.6]**
Shank ROM (°)	71.8 [66.5; 75.4]	67.7 [61.9; 72.6]	− **4.6** **[**− **7.9;** − **1.1]**

Data are reported as median [25th percentile; 75th percentile]

*GCD* gait cycle duration, *ROM* range of motion, *n* number of prostheses

Statistically significant changes between time points are bolded (*p* ≤ 0.05)

## Discussion

The most important findings of the present study were that clinically relevant improvements were observed for all primary measures at the three follow-up time points. Furthermore, *EQ-5D* as well as knee flexion and neutral angles reported statistically significant changes following surgery, also indicating improvements at 1 yr, 5 yr and 10 yr. While the absence of minimum important change (MIC) threshold limited the interpretation of the clinical significance of the improvement in these secondary measures, it is worth mentioning that the changes exceeded the error of measurement [5, 35] and certainly reflect real changes. Interestingly, from all the measures collected at baseline, only one, the maximum extension angle, did not change statistically following surgery. This is probably related to the lower-extremity muscles of the patients and not to the prosthesis itself. Additionally to the positive results in the diverse domains assessed by the questionnaires, the small revision rate (without any revision for loosening) was encouraging regarding the long-term resistance to wear [40].

Besides the evolution compared to baseline discussed in the paragraph hereinabove, it is interesting to note that the present study sporadically reported statistically significant changes among follow-up evaluations. The heterogeneity of these few changes, observed at different time points for different measures, first suggested that the benefits of the arthroplasties were already fully achieved after one year. Nevertheless, these changes, combined with the radiographic and gait evolutions, also underlined a global worsening, of low magnitude between 1 and 10 yr. As reported in the literature, the prevalence of knee pain increases and the mobility decreases with the aging of the population, particularly in the elderly [2, 25, 34, 37]. Therefore, the global worsening observed in the present study is most likely driven by the aging of the participants rather than by the prosthesis [14, 32, 36, 43].

**Fig. 5:**
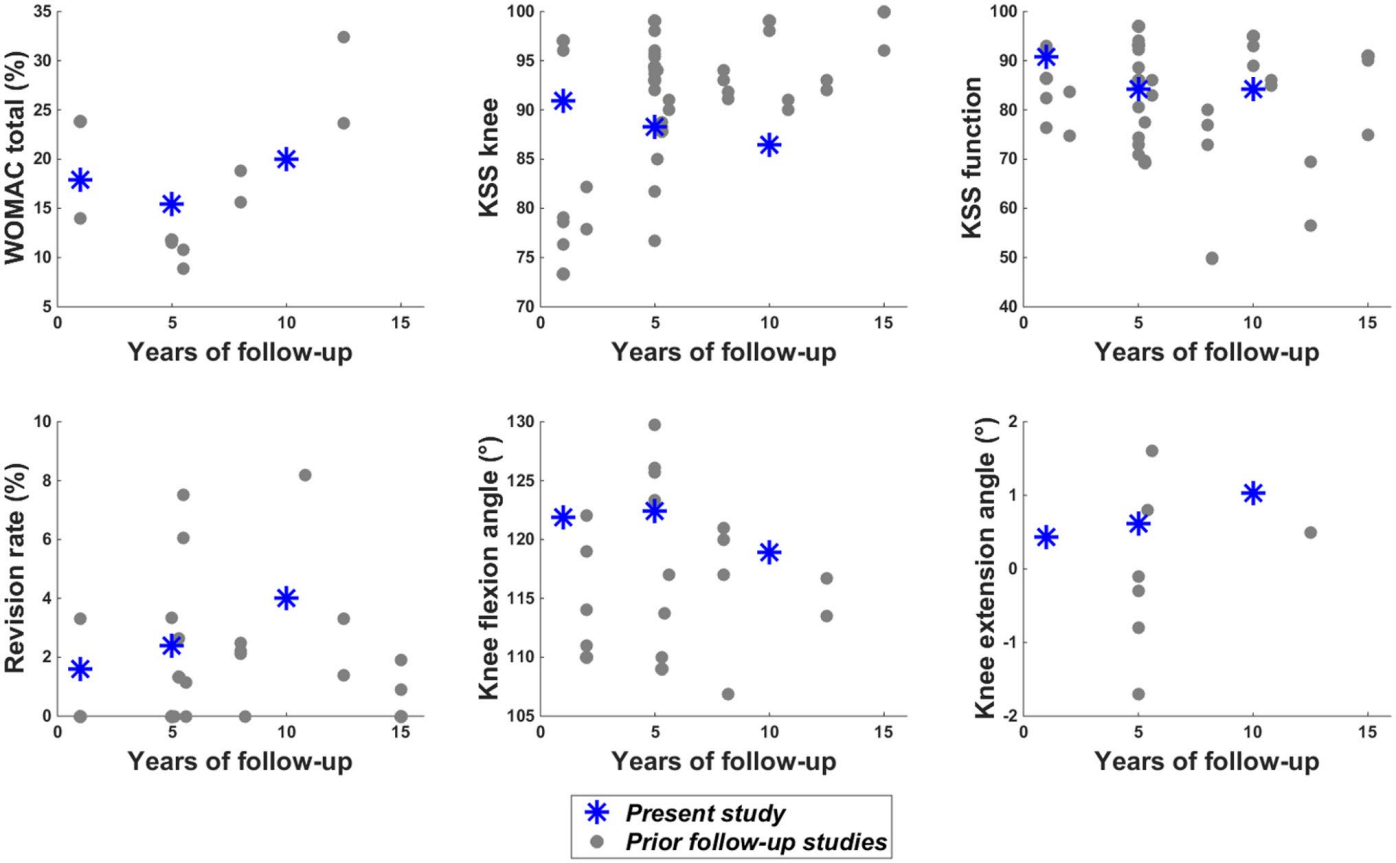
Presentation of the study results (blue stars) for the primary measures with respect to literature (gray dots). The results of 15 prior studies are summarized in this figure [1, 12, 18, 21–26, 30, 38, 39, 41, 42, 44]. Comparison studies were selected based on their inclusion in at least one of three systematic reviews on total knee prosthesis design [6, 19, 28]. *WOMAC* Western Ontario and McMaster Universities Osteoarthritis Index, *KSS* Knee Society score

**Fig. 6:**
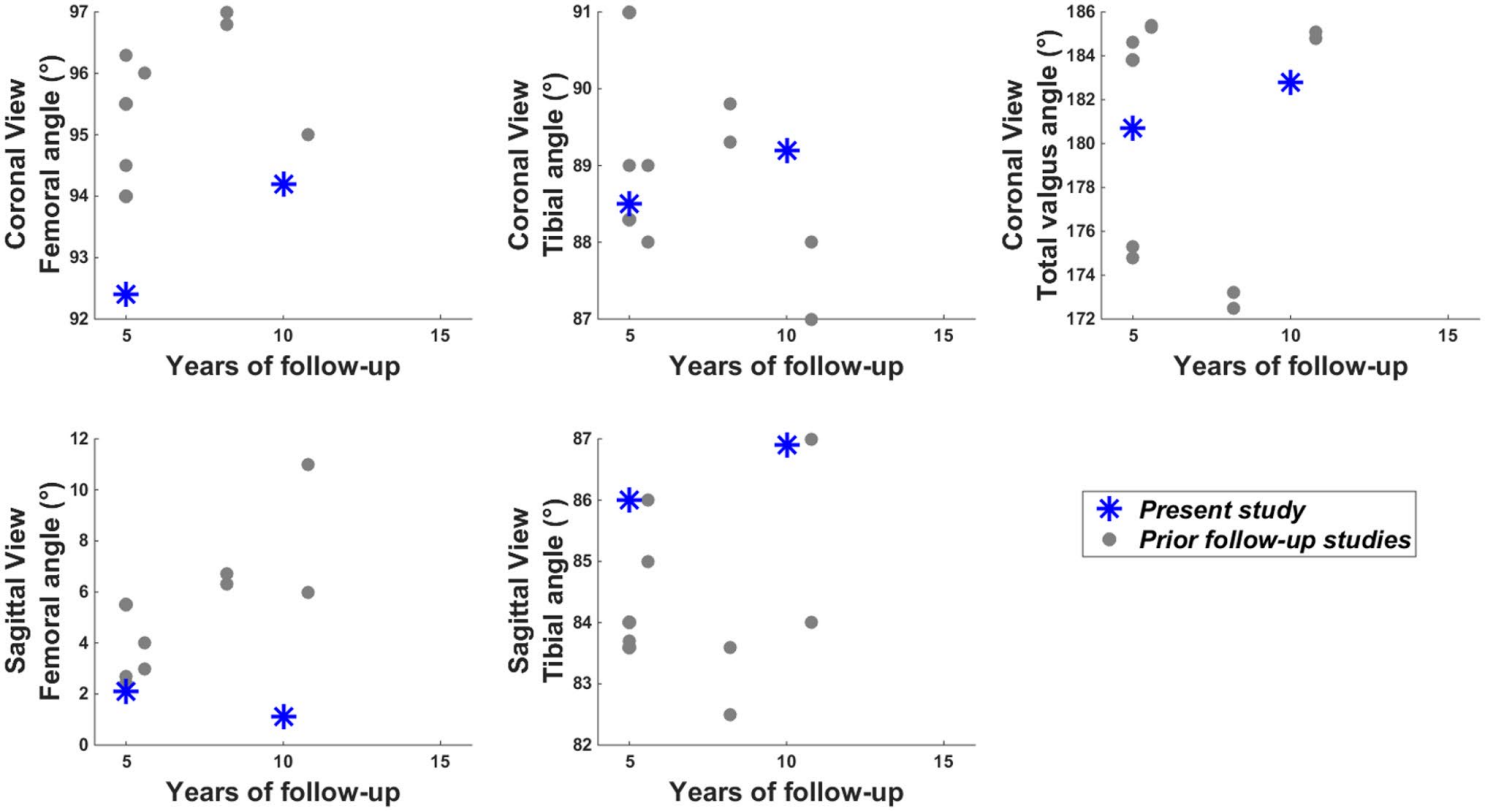
Presentation of the study results (blue stars) for the radiography measures with respect to literature (gray dots). The results of 6 prior studies are summarized in this figure [21, 23, 24, 39, 42, 44]. Comparison studies were selected based on their inclusion in at least one of three systematic reviews on total knee prosthesis design [6, 19, 28]

Based on three systematic reviews on the clinical evolution following total knee arthroplasty [6, 19, 29], fifteen studies were identified with variables of interest similar to those in the present study and at least five-year of follow-up [1, 12, 18, 21–24, 26, 27, 31, 38, 39, 41, 42, 44]. Their results are plotted with those of the present study in Fig. 5 for patient reported outcomes and Fig. 6 for radiography measures. Remaining cautious regarding the comparison of different studies, these plots globally suggest similar results for the FIRST^®^ and the other prosthesis designs. The comparison of the gait results with prior research is extremely limited as, to our knowledge, only one total knee arthroplasty follow-up study included such an analysis [21]. The finding of less effective gait at 5 yr than at 1 yr was nonetheless consistent between this prior publication and the present study assessing prostheses of different design. Consequently, this study demonstrated an effective functioning of the FIRST^®^ prosthesis during the 10 years of observation.

This study has some limitations worth discussing. First, unexpected life events led to the loss of numerous patients at follow-up, in 96% of cases, for reasons unrelated to the study prosthesis. Nevertheless, the number of prostheses evaluated at 10 yr was still more than twice the minimum number from the sample size calculation. In accordance with the study objectives, a general assessment of the FIRST^®^ prosthesis was performed based on all included patients. Further research with sensitivity analyses could nonetheless be interesting, for example to characterize the effect of the patients’ age. Second, radiographic and gait analyses were only performed at some follow-up time points, which limited the characterization of the evolution following surgery. Yet, the time points for radiographic and gait analyses were selected carefully, with respect to prior literature [21, 33] and based on the resources available for the present study, to describe the most relevant properties of the FIRST^®^ prosthesis as well as to allow consolidation with other works.

## Conclusion

The positive results observed in all domains of assessment and the small revision rate demonstrated an effective functioning of the FIRST^®^ prosthesis during the ten-year follow-up period. The results, globally similar to those previously published for other prosthesis designs, encourage the development of assistive tools to select the most appropriate prosthesis on an individual basis.

## Supplementary Information

Below is the link to the electronic supplementary material.

Supplementary file1 (DOCX 26 KB)

## Data Availability

N.A.

## Code availability

N.A.

## References

[1] Akasaki Y, Matsuda S, Miura H, Okazaki K, Moro-oka T-a, Mizu-Uchi H (2009). Total knee arthroplasty following failed high tibial osteotomy: mid-term comparison of posterior cruciate-retaining versus posterior stabilized prosthesis. Knee Surg Sports Traumatol Arthrosc.

[2] Andersen RE, Crespo CJ, Ling SM, Bathon JM, Bartlett SJ (1999). Prevalence of significant knee pain among older Americans: results from the Third National Health and Nutrition Examination Survey. J Am Geriatr Soc.

[3] Bach CM, Biedermann R, Goebel G, Mayer E, Rachbauer F (2005). Reproducible assessment of radiolucent lines in total knee arthroplasty. Clin Orthop.

[4] Bellamy N, Buchanan WW, Goldsmith CH, Campbell J, Stitt LW (1988). Validation study of WOMAC: a health status instrument for measuring clinically important patient relevant outcomes to antirheumatic drug therapy in patients with osteoarthritis of the hip or knee. J Rheumatol.

[5] Brosseau L, Balmer S, Tousignant M, O'Sullivan JP, Goudreault C, Goudreault M (2001). Intra-and intertester reliability and criterion validity of the parallelogram and universal goniometers for measuring maximum active knee flexion and extension of patients with knee restrictions. Arch Phys Med Rehabil.

[6] Cheng M, Chen D, Guo Y, Zhu C, Zhang X (2013). Comparison of fixed-and mobile-bearing total knee arthroplasty with a mean five-year follow-up: a meta-analysis. Exp Ther Med.

[7] Chow S-C, Shao J, Wang H, Lokhnygina Y (2017). Sample size calculations in clinical research.

[8] Churchill D, Incavo S, Johnson C, Beynnon B (2001). The influence of femoral rollback on patellofemoral contact loads in total knee arthroplasty. J Arthroplasty.

[9] Clary CW, Fitzpatrick CK, Maletsky LP, Rullkoetter PJ (2013). The influence of total knee arthroplasty geometry on mid-flexion stability: an experimental and finite element study. J Biomech.

[10] Clement ND, Bardgett M, Weir D, Holland J, Gerrand C, Deehan DJ (2018). What is the minimum clinically important difference for the WOMAC index after TKA?. Clin Orthop.

[11] Dejnabadi H, Jolles BM, Casanova E, Fua P, Aminian K (2006). Estimation and visualization of sagittal kinematics of lower limbs orientation using body-fixed sensors. IEEE Trans Biomed Eng.

[12] Delport H (2013). The advantage of a total knee arthroplasty with rotating platform is only theoretical: prospective analysis of 1,152 arthroplasties. Open Orthop J.

[13] Dolan P (1997). Modeling valuations for EuroQol health states. Med Care.

[14] Elmallah RD, Jauregui JJ, Cherian JJ, Pierce TP, Harwin SF, Mont MA (2016). Effect of age on postoperative outcomes following total knee arthroplasty. J Knee Surg.

[15] Ewald FC (1989). The Knee Society total knee arthroplasty roentgenographic evaluation and scoring system. Clin Orthop.

[16] Gibon E, Mouton A, Passeron D, Le Strat V, Graff W, Marmor S (2014). Doctor, what does my knee arthroplasty weigh?. J Arthroplasty.

[17] Grupp TM, Schroeder C, Kim TK, Miehlke RK, Fritz B, Jansson V (2014). Biotribology of a mobile bearing posterior stabilised knee design-effect of motion restraint on wear, tibio-femoral kinematics and particles. J Biomech.

[18] Harato K, Bourne RB, Victor J, Snyder M, Hart J, Ries MD (2008). Midterm comparison of posterior cruciate-retaining versus-substituting total knee arthroplasty using the genesis II prosthesis: a multicenter prospective randomized clinical trial. Knee.

[19] Hofstede SN, Nouta KA, Jacobs W, van Hooff ML, Wymenga AB, Pijls BG (2015). Mobile bearing vs fixed bearing prostheses for posterior cruciate retaining total knee arthroplasty for postoperative functional status in patients with osteoarthritis and rheumatoid arthritis. Cochrane Database Syst Rev.

[20] Insall JN, Dorr LD, Scott RD, Scott WN (1989). Rationale of the Knee Society clinical rating system. Clin Orthop.

[21] Jolles B, Grzesiak A, Eudier A, Dejnabadi H, Voracek C, Pichonnaz C (2012). A randomised controlled clinical trial and gait analysis of fixed-and mobile-bearing total knee replacements with a five-year follow-up. J Bone Joint Surg Br.

[22] Kalisvaart MM, Pagnano MW, Trousdale RT, Stuart MJ, Hanssen AD (2012). Randomized clinical trial of rotating-platform and fxed-bearing total knee arthroplasty: no clinically detectable differences at 5 years. J Bone Joint Surg Am.

[23] Kim YH, Kim DY, Kim JS (2007). Simultaneous mobile- and fxed-bearing total knee replacement in the same patients. A prospective comparison of mid-term outcomes using a similar design of prosthesis. J Bone Joint Surg Br.

[24] Kim YH, Kim JS (2009). Prevalence of osteolysis after simultaneous bilateral fxed- and mobile-bearing total knee arthroplasties in young patients. J Arthroplasty.

[25] Kirkwood RN, de Souza MB, Mingoti SA, Faria BF, Sampaio RF, Resende RA (2018). The slowing down phenomenon: what is the age of major gait velocity decline?. Maturitas.

[26] Kolisek FR, McGrath MS, Marker DR, Jessup N, Seyler TM, Mont MA, Lowry Barnes C (2009). Posterior-stabilized versus posterior cruciate ligament-retaining total knee arthroplasty. Iowa Orthop J.

[27] Lee SM, Seong SC, Lee S, Choi WC, Lee MC (2012). Outcomes of the different types of total knee arthroplasty with the identical femoral geometry. Knee Surg Relat Res.

[28] Lizaur-Utrilla A, Gonzalez-Parreño S, Martinez-Mendez D, Miralles-Muñoz FA, Lopez-Prats FA (2020). Minimal clinically important diferences and substantial clinical benefts for Knee Society Scores. Knee Surg Sports Traumatol Arthrosc.

[29] Longo UG, Ciufreda M, Mannering N, D’Andrea V, Locher J, Salvatore G (2018). Outcomes of posterior-stabilized compared with cruciate-retaining total knee arthroplasty. J Knee Surg.

[30] Mangin M, Galliot F, Houfani F, Baumann C, Mainard D (2022). Return to work after primary total hip or knee arthroplasty. First French study. Retrospective study of 241 cases. Orthop Traumatol Surg Res.

[31] Matsumoto T, Muratsu H, Kubo S, Matsushita T, Kurosaka M, Kuroda R (2012). Intraoperative soft tissue balance reflects minimum 5-year midterm outcomes in cruciate-retaining and posterior-stabilized total knee arthroplasty. J Arthroplasty.

[32] McCalden RW, Robert CE, Howard JL, Naudie DD, McAuley JP, MacDonald SJ (2013). Comparison of outcomes and survivorship between patients of different age groups following TKA. J Arthroplasty.

[33] McClelland JA, Webster KE, Feller JA (2007). Gait analysis of patients following total knee replacement: a systematic review. Knee.

[34] Milanović Z, Pantelić S, Trajković N, Sporiš G, Kostić R, James N (2013). Age-related decrease in physical activity and functional fitness among elderly men and women. Clin Interv Aging.

[35] Palta M, Chen H-Y, Kaplan RM, Feeny D, Cherepanov D, Fryback DG (2011). Standard error of measurement of 5 health utility indexes across the range of health for use in estimating reliability and responsiveness. Med Decis Making.

[36] Pitta M, Khoshbin A, Lalani A, Lee LY, Woo P, Westrich GH (2019). Age-related functional decline following total knee arthroplasty: risk adjustment is mandatory. J Arthroplasty.

[37] Sakakibara H, Zhu S-K, Furuta M, Kondo T, Miyao M, Sy Y (1996). Knee pain and its associations with age, sex, obesity, occupation and living conditions in rural inhabitants of Japan. Environ Heal Prev Med.

[38] Sando T, McCalden RW, Bourne RB, MacDonald SJ, Somerville LE (2015). Ten-year results comparing posterior cruciate-retaining versus posterior cruciate-substituting total knee arthroplasty. J Arthroplasty.

[39] Shemshaki H, Dehghani M, Eshaghi MA, Esfahani MF (2012). Fixed versus mobile weight-bearing prosthesis in total knee arthroplasty. Knee Surg Sports Traumatol Arthrosc.

[40] van der Voort P, Pijls BG, Nouta KA, Valstar ER, Jacobs WC, Nelissen RG (2013). A systematic review and meta-regression of mobile-bearing versus fixed-bearing total knee replacement in 41 studies. Bone Joint J.

[41] Victor J, Banks S, Bellemans J (2005). Kinematics of posterior cruciate ligament-retaining and -substituting total knee arthroplasty: a prospective randomised outcome study. J Bone Joint Surg Br.

[42] Watanabe T, Tomita T, Fujii M, Hashimoto J, Sugamoto K, Yoshikawa H (2005). Comparison between mobile-bearing and fixed-bearing knees in bilateral total knee replacements. Int Orthop.

[43] Williams DP, Price AJ, Beard DJ, Hadfield SG, Arden NK, Murray DW, Field RE (2013). The effects of age on patient-reported outcome measures in total knee replacements. Bone Joint J.

[44] Yagishita K, Muneta T, Ju YJ, Morito T, Yamazaki J, Sekiya I (2012). High-flex posterior cruciate-retaining vs posterior cruciate-substituting designs in simultaneous bilateral total knee arthroplasty: a prospective, randomized study. J Arthroplasty.

