# Supplementary material for: Ten-year follow-up of a total knee prosthesis combining multi-radius, ultra-congruency, posterior-stabilization and mobile-bearing insert shows long-lasting clinically relevant improvements in pain, stiffness, function and stability
Source: Knee Surg Sports Traumatol Arthrosc. 2022 Nov 6;31(3):1043–52. doi: 10.1007/s00167-022-07216-8 (PMC9958143; doi:10.1007/s00167-022-07216-8)
Supplement: Supplementary file 1 — Supplementary file1 (DOCX 26 KB) [file 167_2022_7216_MOESM1_ESM.docx]

**SUPPLEMENTARY MATERIALS**

**SUPPLEMENTARY MATERIAL 1**

**Table.** Evolution of primary measures between time points.

| MEASURES | EVOLUTION | | | | | | |
| --- | --- | --- | --- | --- | --- | --- | --- |
|  | 1yr – BL  n = 116 | 5yr – BL  n = 88 | 10yr – BL  n = 68 | 5yr – 1yr  n = 85 | 10yr – 1yr  n = 66 | 10yr – 5yr  n = 66 |  |
| WOMAC total (0-100%) | **-29.2**  **[-43.8; -18.2]** | **-32.3**  **[-47.9; -21.4]** | **-30.2**  **[-44.8; -16.1]** | -1.0  [-11.5; 5.2] | 1.6  [-6.3; 8.3] | **4.2**  **[-1.3; 13.5]** |  |
| WOMAC pain (0-100%) | **-40.0**  **[-50.0; -25.0]** | **-45.0**  **[-60.0; -27.5]** | **-37.5**  **[-55.0; -20.0]** | 0.0  [-10.0; 5.0] | 0.0  [-10.0; 10.0] | 5.0  [-5.0; 10.0] |  |
| WOMAC stiffness (0-100%) | **-25.0**  **[-50.0; 0.0]** | **-37.5**  **[-50.0; -12.5]** | **-25.0**  **[-50.0; -12.5]** | **-12.5**  **[-25.0; 0.0]** | 0.0  [-25.0; 0.0] | 0.0  [0.0; 15.6] |  |
| WOMAC function (0-100%) | **-25.7**  **[-44.1; -16.2]** | **-29.4**  **[-47.1; -16.9]** | **-26.5**  **[-43.4; -12.5]** | 0.0  [-8.8; 5.9] | 2.2  [-4.4; 10.3] | **2.9**  **[0.0; 14.7]** |  |
| KSS knee (0-100) | **30.0**  **[19.5; 42.0]** | **28.5**  **[17.0; 43.0]** | **29.5**  **[15.0; 41.0]** | **-2.0**  **[-7.0; 1.3]** | -1.0  [-8.0; 2.0] | 0.0  [-5.0; 7.0] |  |
| KSS function (0-100) | **27.5**  **[20.0; 40.0]** | **20.0**  **[7.5; 30.0]** | **20.0**  **[-20.0; 0.0]** | **0.0**  **[-20.0; 0.0]** | **0.0**  **[-20.0; 0.0]** | 0.0  [-10.0; 0.0] |  |

Data are reported as median [25^th^ percentile; 75^th^ percentile].

WOMAC: Western Ontario and McMaster Universities Osteoarthritis Index. KSS: Knee Society score.

BL: baseline. 1yr, 5yr and 10yr: one, five and ten year follow-ups, respectively.

n: number of prostheses.

Statistically significant changes between time points are bolded (adjusted p ≤ 0.05). Changes that were also clinically significant (median change above MIC) are underlined.

**SUPPLEMENTARY MATERIAL 2**

**Table.** Evolution of EuroQol quality of life (EQ-5D) and passive flexion-extension knee angles between time points.

| MESURES | EVOLUTION | | | | | | |
| --- | --- | --- | --- | --- | --- | --- | --- |
|  | 1yr – BL  n = 116 | 5yr – BL  n = 88 | 10yr – BL  n = 68 | 5yr – 1yr  n = 85 | 10yr – 1yr  n = 66 | 10yr – 5yr  n = 66 |  |
| EQ-5D questionnaire (0-1) | **0.3**  **[0.1; 0.5]** | **0.2**  **[0.0; 0.4]** | **0.2**  **[0.0; 0.4]** | **0.0**  **[-0.2; 0.0]** | **-0.1**  **[-0.3; 0.0]** | 0.0  [-0.2; 0.1] |  |
| EQ-5D VAS (0-100) | **10.0**  **[0.0; 28.0]** | **10.0**  **[-7.5; 21.5]** | **10.0**  **[-5.0; 20.0]** | **-5.0**  **[-20.0; 0.0]** | **-5.0**  **[-17.5; 2.5]** | 0.0  [-10.0; 5.0] |  |
| Flexion angle (°) | **2.5**  **[-7.5; 10.0]** | **5.0**  **[-5.0; 12.0]** | 1.0  [-8.5; 9.0] | 0.0  [-6.3; 5] | -4.0  [-9.0; 5.0] | **-2.5**  **[-7.0; 1.0]** |  |
| Neutral angle (°) | **0.0**  **[0.0; 0.0]** | **0.0**  **[-8.0; 0.0]** | **0.0**  **[-7.5; 0.0]** | 0.0  [0.0; 0.0] | 0.0  [0.0; 0.0] | 0.0  [0.0; 0.0] |  |
| Extension angle (°) | 0.0  [0.0; 0.0] | 0.0  [0.0; 0.0] | 0.0  [0.0; 1.5] | 0.0  [0.0; 0.0] | **0.0**  **[0.0; 2.0]** | 0.0  [0.0; 0.0] |  |

Data are reported as median [25^th^ percentile; 75^th^ percentile].

BL: baseline. 1yr, 5yr and 10yr: one, five and ten year follow-ups, respectively.

n: number of prostheses.

Statistically significant changes between time points are bolded (adjusted p ≤ 0.05).
